# Russell’s viper envenomation: The challenge of diagnosis

**DOI:** 10.37796/2211-8039.1649

**Published:** 2025-06-01

**Authors:** Jing-Hua Lin, Wang-Chou Sung, Jiunn-Wang Liao, Dong-Zong Hung

**Affiliations:** a Division of Toxicology, China Medical University Hospital, Taichung, Taiwan; b National Health Research Institutes, National Institute of Infectious Diseases and Vaccinology, Miaoli, Taiwan; c Graduate Institute of Veterinary Pathobiology, National Chung Hsing University, Taichung, Taiwan

**Keywords:** Snake bite, Russell’s viper, Consumption coagulopathy, Envenomation

## Abstract

Russell’s viper envenomation is rare in Taiwan, and its typical clinical presentations, including consumption coagulopathy, acute renal failure, haemolysis, and increased capillary permeability, have been reported in the literature as case reports or series. Here, we report a case with an atypical presentation, and suspected to be a Russell’s viper bite due to the distinct distribution characteristics of the snake and some progressive clinical signs/symptoms. He returned to health successfully after the correct antivenom was administered, and envenomation was ultimately confirmed by venom detection in the patient’s serum and urine samples.

## 1. Introduction

Russell’s viper is a common venomous snake in Asia that induces severe injuries in humans, so-called envenomation; in some countries or regions, it is one of the most medically important snake species [1]. In Southeast Asia, Russell’s viper snakebite envenomation mostly causes severe coagulation abnormalities, bleeding, and acute renal failure [2]. In addition to haematologic manifestations, neurotoxicity causes muscle weakness [3]. Therefore, the manual published by the World Health Organization specifically mentions that Russell’s viper bites should be differentiated from cobra bites. In fact, Russell’s viper bites must also be distinguished from other common haemorrhagic snakebites in Southeast Asia, especially those caused by *Calloselasma rhodostoma* or *Trimeresurus spp.* [4].

Russell’s viper bites are uncommon in Taiwan, and past field investigations have revealed that these snakes mainly inhabit mountainous areas or specific terrain areas, such as reclaimed land and riverbed gravel flats, and are restrictively distributed in the eastern and southernmost tips of Taiwan [5]. Because the clinical manifestations of coagulation dysfunction and local erythematous swelling caused by Russell’s viper envenomation are similar to those of other viper envenomation, Russell’s viper bites often result in diagnostic delay and lead to serious consequences [6]. Here, we report a case of a patient who was bitten by a Russell’s viper without a witness; the early diagnosis was confusing, and the diagnosis was ultimately confirmed by laboratory tests and successful treatment. This case illustrated that venomous snakebites might occur in some specific areas of Taiwan and may cause coagulation dysfunction, that emergency physicians must consider the diagnosis of Russell’s viper bites.

## 2. Case report

A 41-year-old male weighing 100 kg was bitten on his right foot by an unknown snake species. While walking on the beach of a resort at the southernmost tip of Taiwan in the evening. He was sent to a local hospital immediately. A nonspecific insect bite was observed by the emergency doctor due to only mild erythematous swelling of the right foot, and observation at the hotel was advised. The patient rushed to his home approximately 300 km from the resort the next morning due to pain, progressive swelling, and the development of ecchymosis on the right thigh. He was referred to our emergency room 22 h after the snakebite.

**Fig. 1:**
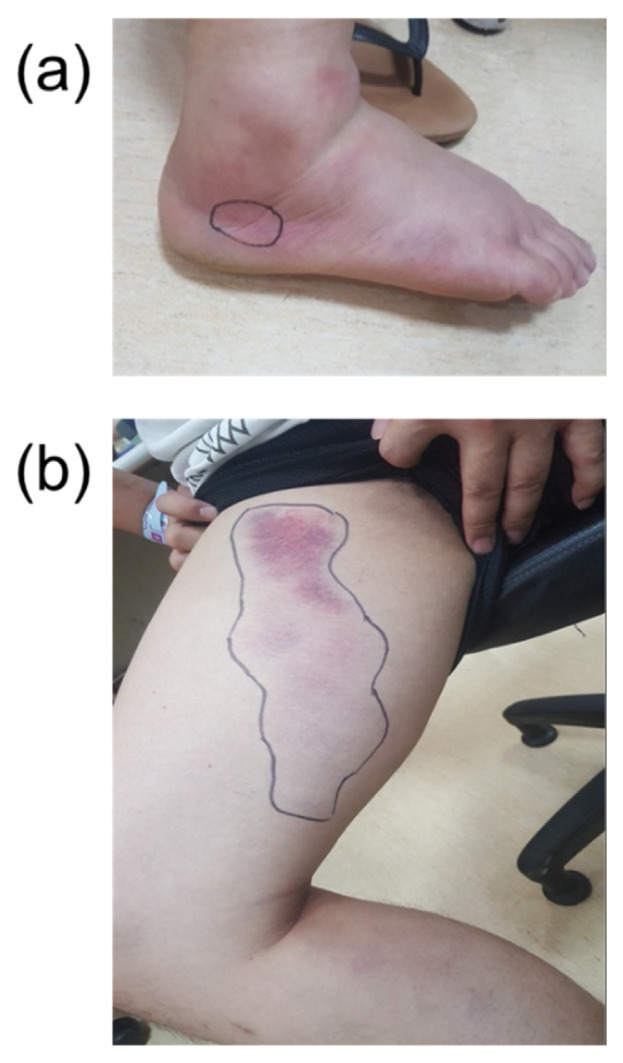
(a) Right foot. Two fang marks were found below the right ankle (marked by a blue circle), with significant swelling and multiple sites of ecchymosis over the right lower leg. (b) A large area of subcutaneous ecchymosis and bruising over the right thigh.

The patient was clearly conscious but in an acute, ill-looking state; his blood pressure was 167/114 mmHg, and heart rate 110/min initially. During hospitalization, the patient’s blood pressure returned to normal, but erythematous swelling of the right foot with subcutaneous ecchymosis occurred, and a large area of bruising over the right thigh was observed (Fig. 1). Laboratory examination revealed normal liver function, renal function, and electrolytes; a normal prothrombin time (PT) and activated partial thromboplastin time (aPTT); and trace occult blood in the urine. Other laboratory data were as follows: platelet count: 118 × 10^3^/μl (normal: 130–400 × 10^3^/μl); fibrinogen level: 175.1 mg/dL (normal: 200–400); D-DIMER level (ELISA): 701.78 ng/mL (FEU) (cut-off value < 500 ng/mL (FEU); and D-Haptoglobin level: 66.0 mg/dL (normal: 36–195)). Because the patient had mild thrombocytopenia, had a slightly high D-DIMER level and had been bitten by a snake at the southernmost tip of Taiwan, a Russell’s viper bite was highly suspected. Two vials of antivenom specific to Russell’s viper were finally administered approximately 32 h after the snakebite due to its shortage as an orphan drug in Taiwan. The patient’s right leg improved after antivenom treatment, and his serum fibrinogen level returned to normal on the third day. However, gastrointestinal (GI) discomfort with nausea, abdominal pain, and loose stool (diarrhoea) occurred on that day. The GI tract manifestations had been noted to be one of the most important clinical features before [2,6]. Three additional vials of antivenom were administered on the 5th day and 8th day due to persistent thrombocytopenia and watery diarrhoea. Eventually, the patient’s platelet count returned to normal, and he no longer experienced diarrhoea on the 9th day. He was discharged with a change in the colour of the residual skin bruising after 10 days of hospitalization and was in good condition at the outpatient clinic follow-up 2 weeks later. Russell’s viper bite was subsequently confirmed by ELISA, and 165.3 ng/ml of venom was detected in the patient’s serum.

## 3. Discussion

At present, in various regions or countries worldwide, the determination of snake species in cases of venomous snake bites still rely on a syndromic approach unless the patient has the snake specimen, whether alive or dead, or presents a photograph. In cases of unwitnessed snake bites, the correct diagnosis and use of specific antivenoms are delayed and might be complicated by worsening clinical features [4], as in the case described here. Taiwan is a small island located in a subtropical region, and according to the 2016 WHO guidelines for the manufacturing of snake antivenom, there are six medically important species of venomous snakes in Taiwan [7]. In clinical practice, the correct diagnosis and treatment of snake bites can be achieved by considering the location of the bite, the appearance of the snake, or typical clinical presentations. The phenotypes of these six venomous snakes are unique and different, and it is not difficult to distinguish them if the bite was witnessed or a clear picture of the snake was available. However, the staggered oval brown patches on the skin of the Russell’s viper snake and the more aligned brown patches on the skin of *Protobothrops mucrosquamatus* (PM) are sometimes not easily distinguished. Fortunately, PM snake envenomation typically results in milder haemorrhagic signs/symptoms such as local redness and bruising, while systemic envenomation from Russell’s viper often leads to rare systemic bleeding, coagulation abnormalities, and decreased platelet counts [2,6,8]. Therefore, this case was certainly distinctive, as it was not caused by a PM snake bite.

There are two other vipers that induce haemorrhagic conditions in Taiwan: one is *Deinagkistrodon acutus* (DA), which bites rarely, and the other is the green viper *Trimeresurus stejnegeri* (TS), which is the most common venomous snake in Taiwan. Envenomation caused by TS bites tends to cause subcutaneous bruising with rare systemic bleeding, coagulation abnormalities or vital organ injuries [8,9]. DA is distributed mainly in the mountainous areas of Taiwan, and its venom might induce bleeding diathesis of wound oozing, subcutaneous haemorrhage, abnormal coagulation function, severe thrombocytopenia and potential regional tissue destruction [10,11], which are not easy to differentiate from the haemorrhagic characteristics induced by Russell’s viper venom. One study revealed that three indicators, including haemorrhagic blisters on the skin, a low platelet count, and a disproportionately high amount of D-dimer in the blood, may help to identify DA envenomation [12]. However, these indicators did not seem to work in this patient with only mildly elevated D-dimer levels; instead, the scene where the patient was bitten while walking on the beach indicated that the offending creature might have been the Russell’s viper.

Furthermore, the clinical manifestations of this patient differed from those of typical cases of Russell’s viper bites reported in the literature [2,6,12,13]. He presented without acute renal failure, haemolysis, rhabdomyolysis, or a prolonged prothrombin time, despite the venom concentration in his serum being as high as 165.3 ng/ml nearly 24 h after being bitten. He presented with only late gastrointestinal symptoms/signs that might have been caused by increased capillary permeability [2,6,14], a mild but persistent platelet count reduction, and slight consumption coagulopathy. Abdominal pain, in addition to coagulopathy, neurotoxicity, and acute renal injury, has been suggested as an early clinical manifestation for distinguishing Russell’s viper snake bites from other viper snakebites in Sri Lanka [15]. But, abdominal discomfort has been noted to be one of significant presentations in cases of cobra (Naja atra) snakebites with severe envenomation in Taiwan [5,16]. The absence of neuropathy and the presence of mild thrombocytopenia and consumption coagulopathy can help rule out the possibility of cobra snake envenoming. Additional doses of antivenom were effective and proved the concept. Based on these findings, identifying atypical but continuously deteriorating clinical manifestations is a substantial challenge for clinicians who can only treat snakebites via a syndromic approach [14]. ICT-Viper, a simple rapid test developed in our previous study, was also used in this case and showed positive results [17]. Laboratory diagnostic methods, enzyme immunoassays or useful rapid tests might help physicians treat patients correctly and in a timely manner to minimize potential injuries or complications from snake toxins.
